# A Joint Johnson–Cook-TANH Constitutive Law for Modeling Saw-Tooth Chip Formation of Ti-6AL-4V Based on an Improved Smoothed Particle Hydrodynamics Method

**DOI:** 10.3390/ma16124465

**Published:** 2023-06-19

**Authors:** Weilong Niu, Yuanpeng Wang, Xuan Li, Ran Guo

**Affiliations:** 1 School of Rail Transportation, Soochow University, Suzhou 215137, China; wlniu@suda.edu.cn (W.N.); ypwang0516@suda.edu.cn (Y.W.); 2 School of Mechanical and Electrical Engineering, Soochow University, Suzhou 215137, China

**Keywords:** titanium alloys, smoothed particle hydrodynamics (SPH), JC-TANH constitutive law, saw-tooth chip

## Abstract

Titanium alloy is a crucial structural material in the modern aerospace field due to its strong corrosion resistance and strength, low density, and reduced sensitivity to vibration load and impact load, as well as its ability to resist expansion in the case of cracks. However, during high-speed cutting of titanium alloy, it is prone to periodic saw-tooth chip formation, which can cause high-frequency fluctuations in the cutting force, aggravate the vibration of the machine tool system, and ultimately reduce the tool’s service life and the workpiece’s surface quality. In this study, we investigated the influence of the material constitutive law in modeling the Ti-6AL-4V saw-tooth chip formation and proposed a joint material constitutive law JC-TANH which was developed based on the Johnson–Cook constitutive law and the TANH constitutive law. It has two advantages of the two models (JC law and TANH law), which means that it can describe the dynamic properties accurately, the same as the JC model, not only under low strain but also under high strain. The most important thing is that it does not need to fit the JC curve at the early stage of strain changes. Additionally, we established a developed cutting model, which integrates the new material constitutive, and the improved SPH method to predict chip morphology, cutting and thrust forces which are collected by the force sensor; we also compared the data with experimental results. Experimental results show that this developed cutting model can better explain the shear localized saw-tooth chip formation and correctly estimate its morphology as well as the cutting forces.

## 1. Introduction

High-speed precision cutting technology is the key of manufacturing technology, reflecting the level of a country’s machinery industry. With the booming development of the aerospace industry, titanium alloy has become an indispensable structural material in modern aerospace field due to its strong corrosion resistance, high strength, low sensitivity to vibration and impact loads, and low expansion in the presence of cracks in the material. It has also been widely used in shipbuilding, medical, and automotive industries [1]. During high-speed cutting of titanium alloys, however, periodic saw-tooth-shaped chips are easily formed, and the formation of saw-tooth-shaped chips can cause high-frequency fluctuations in cutting force, exacerbating the vibration of the machine tool system, thereby reducing the tool life and surface quality of the workpiece. Therefore, in-depth research on the evolution mechanism of saw-tooth-shaped chips is conducive to scientifically revealing the formation process of saw-tooth-shaped chips, extending tool life, optimizing cutting parameters, improving processing technology, determining chip-breaking parameters, and controlling the machining quality and chip morphology of the workpiece surface, which is of great significance to practical production.

Titanium alloy processing mainly faces two problems: (1) high processing cost; (2) low machining accuracy and surface quality caused by improper selection of cutting parameters. Studying the cutting mechanism of titanium alloys helps people understand the wear mechanism of cutting tools, guide the geometric design of cutting tools, and reasonably select cutting dosage while providing reliable reference data for optimizing cutting parameters and achieving the goal of saving processing costs and improving processing quality.

Finite Element Method (FEM) as the most important numerical method for simulating engineering problems has been adopted by most scholars who study cutting problems [2,3,4,5]. Many scholars have conducted a lot of research on the simulation of saw-toothed chip morphology. Rhim [6] proposed a new flow stress model to predict the saw-toothed chip morphology of the AISI1045 material. Sowerby et al. [7] obtained saw-toothed chips through experimental testing and determined that their formation was caused by uneven plastic deformation, proposing a slip-line method to evaluate the accumulation of internal deformation and damage of materials during the processing and simulate the formation of saw-toothed chips. Su et al. [8] studied the process and mechanism of saw-toothed chip formation during high-speed cutting based on the plastic–metal material’s plastic–brittle transformation, revealing the saw-toothed chip morphology’s effect on the cutting process and tool wear. Shivpuri et al. [9,10] proposed the mechanical conditions for the formation of serrated chips during high-speed cutting and established a predictive model for the critical cutting conditions for chip serration based on the thermo-mechanical coupling of material in the first deformation zone. In addition, P. Fernandez-Zelaia, G. Liu, T. Thepsonthi, F. Wang, D. Setti, F.R. Klocke, and others have also established cutting models for the formation of serrated chips [11,12,13,14,15,16].

Some scholars have also conducted some research on tool coatings, tool shape and cutting tool angle in the cutting process. By numerical simulation analysis, Kukielka, L found that the tool shape and cutting angle play a key role in the cutting process. These factors affect cutting force, cutting temperature, surface roughness, workpiece stress state and tool wear rate. The shape of the tool directly affects the cutting process. For example, the radius of the tip affects cutting force and cutting temperature. Tool shape also affects chip formation and removal, which has a direct effect on tool wear and workpiece surface quality. Cutting angle also has a significant effect on cutting force, cutting temperature, and chip formation. The change in cutting angle changes the contact area during the cutting process, thus affecting the cutting force and cutting temperature, and further affecting the states of strain and stress in the surface layer of object. The cutting angle also affects the contact area between the tool and the workpiece, which affects the rate of tool wear [17]. Some scholars have also conducted some work on tool coatings to improve the cutting tool performance. Sahu et al. provided a novel method for altering the volume fraction of TiN and tailoring the microstructure of the investigated Ni1-xTixN nanocomposite films by varying the Ti (RF) target power to obtain a desirable combination of surface properties required in protective coatings [18].

However, since the FEM is a numerical method based on a mesh, the entire problem domain is divided into a finite number of meshes in simulating some numerical problems. Therefore, when dealing with material large deformation problems such as high-speed collisions and metal cutting, the excessive distortion of the mesh may cause a decrease in simulation accuracy or even interruption of the calculation.

Therefore, many researchers have set up specific treatment methods in the large deformation region to overcome this problem, such as using adaptive remeshing grid methods, deleting elements, or setting separation criteria between chips and workpiece. However, these methods have drawbacks. Adaptive remeshing grids increase the computation time of the model; deleting grids removes a portion of the workpiece’s mass, which does not conform to reality, and setting separation criteria increases the complexity of modeling. Smoothed Particle Hydrodynamics (SPH) is a non-element-based meshless Lagrangian method [19]. It can be used to get rid of the shackles of mesh, realize the natural classification of chips and workpieces without the setting of separation criteria, and can simulate the ultimate deformation of materials in the process of saw-tooth formation [20,21]. Based on this advantage, this paper uses the SPH numerical method to simulate the cutting process. However, the traditional SPH algorithm cannot accurately reproduce the linear function of the entire problem domain. In the initial form of the particle summation formula, traditional SPH cannot even accurately reproduce constants near the boundary due to the loss of symmetry in smoothing operations. Therefore, an improved SPH algorithm is always necessary. This paper uses an improved SPH algorithm to simulate the entire cutting process by incorporating density correction and kernel gradient correction [22,23].

The solving capability and accuracy of the cutting numerical model are not only related to the physical properties of the material itself, but also have a great relationship with the material’s constitutive model. The constitutive model is an important basis for studying metal deformation, describing the dynamic mechanical properties of materials during the deformation process and reflecting the relationship between stress and strain inside the material after being subjected to compressive deformation under external forces. The Johnson–Cook (JC) model, proposed by Johnson and Cook in the 1980s, is one of the most widely used metal constitutive models to date [24]. It comprehensively considers factors such as temperature, strain rate, and strain; it is simple in form and has a clear physical interpretation. However, this model has insufficient description of materials in the strain-softening stage and is also not suitable for many ductile materials. In order to explain the material’s deformation characteristics when experiencing high strain and failure, Johnson and Cook established the JC damage (failure) model based on the original JC model [25]. They set up a damage rule; when the cumulative damage of the material reaches a certain specific value, the material is considered to have failed. Many scholars have made improvements to the JC model. Mohammad Sima and Madalina Calamaz modified the temperature term, stress term, and strain term of the JC constitutive model and proposed a new constitutive model, which well describes the mechanical properties of materials in the strain-softening stage.

In this work, we proposed a joint constitutive law which joined the JC law and the TANH law together. This work builds on the previous work of the authors and further analyses the chip morphology at different cutting depths [26]. It has two advantages of the two models (JC law and TANH law) and can describe the dynamic properties accurately, the same as the JC model, not only under low strain but also under high strain. We assumed that chip deformation is caused by adiabatic shearing and employed the joined material constitutive law JC-TANH to describe the dynamic mechanical properties of materials. The constitutive law is employed by our in-house SPH code. In addition, two cases with different cutting conditions are implemented to validate the developed cutting model by the comparisons of the experimental chip morphology results and simulation results. To summarize, we adopted three measurements to improve the precision of the Ti-6AL-4V cutting model: (1) A new constitutive material law is applied in this cutting model, which made the strain–stress curve more accurate by integrating the two laws; the time-consuming process of fitting the TANH law to the JC law in the strain-hardening part is avoided and improves the modeling efficiency; in addition, we assured the accuracy of the modeling flow stress by applying the JC law under the low-strain condition; (2) The cutting model does not apply FEM which most of cutting model applied. Without FEM algorithms, the mesh distortion does not occur, and the saw-tooth chip morphology (large deformation) can be obtained exactly; (3) The traditional SPH algorithms are improved by adding modified scheme density correction and kernel gradient correction. The result shows that this improved cutting model can predict the cutting forces and saw-tooth chip morphology more accurately, and it can also explain the chip formation process well during cutting.

## 2. Materials and Methods

### 2.1. Modeling of SPH

#### 2.1.1. SPH Formulation for Solid Flows

The cutting process is a process in which a tool collides and compresses the workpiece at high speed, which causes extreme deformation of the material. Therefore, it is generally difficult to accurately describe using traditional mesh methods such as FEM. On the contrary, the SPH method has advantages in this aspect. Because the model is composed of particles, the mesh distortion does not occur. However, the traditional SPH method usually does not consider the physical strength of the medium and material. Therefore, the physical strength of the material and medium can be taken into account by introducing dynamic control equations with material strength and deriving the solution of the control equations in SPH. In the SPH equation, the governing equation is the Lagrangian form of Navier–Stokes equations (NS equations) [19], and we use it for governing the to-be-cut materials by the mass conservation law and the momentum conservation laws:

(1) $$\frac{d\rho }{dt}=-\rho \frac{\partial v^{\alpha }}{\partial x^{\alpha }},$$

(2) $$\frac{dv^{\alpha }}{dt}=-\frac{1}{\rho }\frac{{\partial \sigma }^{\alpha \beta }}{\partial x^{\beta }}+f^{\alpha }.$$

The system is characterized by the material density (ρ), velocity (v), time (t), and position coordinates (*x*, *y*, *z*). The acceleration of the system is caused by external forces ($f^{\alpha }$), and the total stress tensor ($\sigma^{\alpha \beta }$) also affects the acceleration. In particular, the acceleration component along each coordinate direction (*x*, *y*, *z*) is determined by the net force acting on the system in that direction, which includes both the external forces and the stress tensor contributions. Thus, by considering the forces and stresses acting on the system, one can model the dynamic behavior of the material during the cutting process.

There are two steps in obtaining an SPH formulation [19]. This computation is conducted by performing a simple summation of the contributions from the neighboring particles which are weighted using a kernel function. The kernel function determines the extent of influence of each particle on its neighbors, and it is typically chosen to have a compact support that falls off smoothly to zero at some distance from the particle. By summing up the contributions from all the neighboring particles, we obtain an approximation to the field function and its derivatives at each particle location. In the particle approximation, the computation domain is discretized with a set of particles. The field function and its derivative at particle i can then be a simple summation of the form:

(3) $$<f\left(x_{i}\right)>=\sum_{j=1}^{N}\frac{m_{j}}{\rho_{j}}f(x_{j})W_{ij},$$

(4) $$<\nabla f\left(x_{i}\right)>=\sum_{j=1}^{N}\frac{m_{j}}{\rho_{j}}f(x_{j})\nabla_{i}W_{ij}.$$

The equation involves the positions of particles in the computation domain, denoted by $x_{i}$ and $x_{j}$; the mass of each particle is represented by m, and its density is given by ρ; the equation aims to compute the value of a field function, denoted by $f\left(x\right)$, at each particle location.

The Navier–Stokes equations describe the motion of fluid particles in a continuous domain. However, in numerical simulations, the continuous domain is discretized into a finite number of particles. To model the fluid flow in this discrete domain, the field functions in the Navier–Stokes equations at each particle location are approximated using a kernel function that depends on the spatial distribution of the particles. The momentum equation (Equation (1)) and continuity equation (Equation (2)) are the governing equations of fluid motion. To account for the discrete nature of the domain, these equations can be expressed in terms of the particle-based approximation of the field functions. Specifically, the momentum equation and continuity equation can be written as follows:

(5) $$\left\{\begin{array}{c} \frac{d\rho_{i}}{dt}=-\rho_{i}\sum_{j=1}^{N}\frac{m_{j}}{\rho_{i}}\left(v_{i}^{\beta }-v_{j}^{\beta }\right)\frac{\partial W_{ij}}{\partial x_{i}^{\beta }}\\ \frac{dv_{i}^{\alpha }}{dt}=-\sum_{j=1}^{N}m_{j}\left(\frac{\sigma_{i}^{\alpha \beta }}{\rho_{i}^{2}}+\frac{\sigma_{j}^{\alpha \beta }}{\rho_{j}^{2}}+\Pi_{ij}\right)\frac{\partial W_{ij}}{\partial x_{i}^{\beta }}+f_{i}^{\alpha }\\ \frac{de_{i}}{dt}=-\frac{1}{2}\sum_{j=1}^{N}m_{j}\left(\frac{P_{i}}{\rho_{i}^{2}}+\frac{P_{j}}{\rho_{j}^{2}}+\Pi_{ij}\right)\left(v_{i}^{\beta }-v_{j}^{\beta }\right)\frac{\partial W_{ij}}{\partial x_{i}^{\beta }}+\frac{1}{\rho_{i}}\tau_{i}^{\alpha \beta }\varepsilon_{i}^{\alpha \beta }\\ \frac{dx_{i}^{\alpha }}{dt}=v_{i}^{\alpha } \end{array}\right.,$$

where term $\Pi_{ij}$ is called artificial viscosity. It is a numerical technique that can model shock waves and reduce unphysical oscillations in the numerical results around the shocked region. In this paper, we use the Monaghan-type artificial viscosity, which is a commonly used formulation for Smoothed Particle Hydrodynamics (SPH) simulations [27].

#### 2.1.2. Geometric Definition of Cutting Model in SPH Method

**Figure 1:**
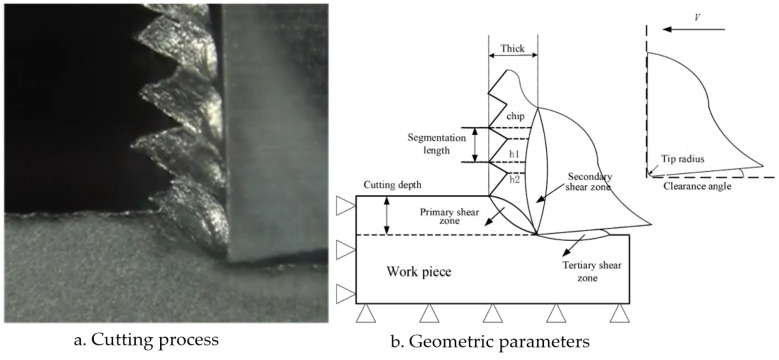
Cutting model.

**Figure 2:**
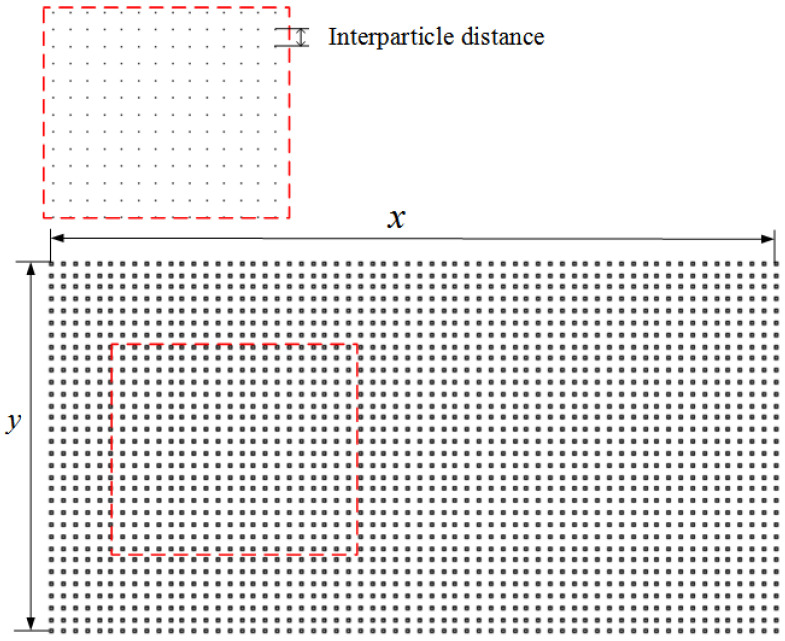
Workpiece particle model (The red square in the figure represent the local particles).

In the process of studying the cutting mechanism, most scholars focus on orthogonal cutting (the cutting edge is perpendicular to the cutting speed direction) because its geometric relationship is relatively simple, and traditional turning and milling can be approximated by coordinate transformations and product transformations from orthogonal cutting. Therefore, orthogonal cutting can be used to explain the cutting mechanism of titanium alloy. The main focus of this study is to establish a numerical model of two-dimensional orthogonal cutting of titanium alloy based on SPH. Figure 1 shows the experimental diagram of the orthogonal cutting model and the definition of parameters in the model (cutting speed *V,* rake angle γ, clearance angle β, height of saw-tooth chip h1, h2, width of saw-tooth chip w, and undeformed chip thickness *u*):

In the SPH numerical method modeling, the model (workpiece model and tool model) needs to be designed, imported, and then particleized. The model established in this study is a 2D model, and the workpiece geometry is rectangular. In the initial design process of the model, the length and height of the model are given, and the particle spacing is set. The particles are uniformly arranged inward from the boundary of the workpiece model, and the entire workpiece model is particleized as shown in Figure 2.

**Figure 3:**
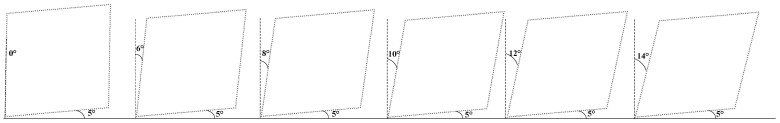
Geometric model of the tool.

**Figure 4:**
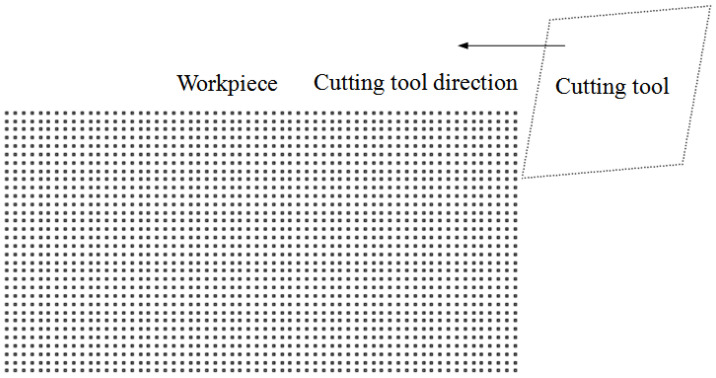
SPH cutting geometry model.

For the geometry of the tool, only the front and back faces of the tool come into contact with the workpiece, as long as the rake angle and clearance angle of the tool are consistent with the design during the cutting process. Therefore, in order to facilitate the modification of the rake angle and clearance angle in the model without affecting the distribution of particles on the entire tool and avoiding remodelling the geometry of the tool, this study uses a quadrilateral as the geometry of the tool. When modifying the rake angle and clearance angle, it is assumed that the geometry of the tool is a quadrilateral, and the length and width of the tool remain unchanged. The number of particles forming the tool remains the same. Figure 3 shows the tool designs with different rake angles. Similarly, the tool design with the clearance angle is the same as that of the rake angle. Figure 4 shows the final SPH cutting geometry model.

#### 2.1.3. Improved Algorithms (Density and Kernel Gradient Correction)

While SPH is capable of handling large deformation problems, the traditional method has limited accuracy in reproducing quadratic and linear functions. As a result, various approaches have been proposed to improve the algorithm, including addressing particle inconsistency at surface boundaries and improving gradient approximations [28,29]. Some researchers have even developed new smoothing functions to satisfy discretized consistency conditions [30]. These methods, however, are often unsuitable for hydrodynamic simulations due to their non-symmetric, non-monotonic, and partially negative characteristics. In this paper, we utilized an enhanced SPH method that has demonstrated effectiveness in simulating solid problems such as cutting processes [22,31]. Compared to traditional SPH, this approach corrects density and kernel gradient using Moving Least Squares and a correction matrix. Specifically, the density correction scheme uses MLS to periodically correct the density field [32], enabling the exact reproduction of linear density variation and smoother pressure. This correction scheme can be expressed as follows:

(6) $$<\rho_{i}>=\sum_{j}^{N}\rho_{j}W_{ij}^{MLS}\frac{m_{j}}{\rho_{j}}=\sum_{j}^{N}m_{j}W_{ij}^{MLS},$$

where $W_{ij}^{MLS}$ is the MLS kernel.

This paper applies the KGC scheme to enhance the computational accuracy of the SPH approximation as described in reference [23]. The KGC scheme offers a straightforward implementation procedure, which involves replacing the kernel gradient $\nabla_{i}W_{ij}$ (as given by Equation (5)) with the corrected kernel gradient. This leads to the restoration of the second-order accuracy of the SPH approximation. The corrected value of the kernel gradient for any given particle *i* is expressed using the following equation:

(7) $$\nabla_{i}^{new}W_{ij}=L{\left(r_{i}\right)}^{-1}\nabla_{i}W_{ij},$$

(8) $$L\left(r_{i}\right)=\sum_{j}\left[\begin{array}{c} x_{ij}\frac{\partial W_{ij}}{\partial x_{i}},y_{ji}\frac{\partial W_{ij}}{\partial x_{i}}\\ x_{ji}\frac{\partial W_{ij}}{\partial y_{i}},y_{ji}\frac{\partial W_{ij}}{\partial y_{i}} \end{array}\right]V_{j}.$$

#### 2.1.4. Constitutive Law for Materials with Strength

To model the behavior of a solid material in a dynamic velocity field with a spatial gradient, the SPH method requires a constitutive equation to connect the internal stresses and pressure of the material with its velocity. This constitutive equation is used to express the total stress tensor $\sigma^{\alpha \beta }$ in Equation (5), which consists of two parts:

(9) $$\sigma^{\alpha \beta }=-P\delta^{\alpha \beta }+\tau^{\alpha \beta },$$

where $\tau^{\alpha \beta }$ is the deviatoric shear stress, P is pressure, and $\delta^{\alpha \beta }$ is the Kronecker tensor. The motion equation can be rewritten as

(10) $$\frac{dv_{i}^{\alpha }}{dt}=-\sum_{j=1}^{N}m_{j}\left(-\left(\frac{P_{i}}{\rho_{i}^{2}}+\frac{P_{j}}{\rho_{j}^{2}}\right)\delta^{\alpha \beta }+\frac{\tau_{i}^{\alpha \beta }}{\rho_{i}^{2}}+\frac{\tau_{j}^{\alpha \beta }}{\rho_{j}^{2}}+\Pi_{ij}\right)\frac{\partial W_{ij}^{new}}{\partial x_{i}^{\beta }}+f_{i}^{\alpha }.$$

The pressure P can be expressed as follows:

(11) $$P=\frac{\rho_{0}c_{0}\eta (1+(1-\frac{\Gamma_{0}}{2})\eta )}{\left(1-(S_{a}-1)\eta \right)}+\rho_{0}\Gamma_{0}e,$$

where

(12) $$\eta =\frac{\rho }{\rho_{0}}-1.$$

$\rho_{0}$ is the (initial) reference density, ρ is actual density, e is the internal energy per unit of mass, $\Gamma_{0}$ is Gruneisen’s gamma at the reference state, $S_{a}$ is the linear Hugoniot slope coefficient, $c_{0}$ is the constant in the linear relation between the shock velocity and particle velocity.

By utilizing the Jaumann rate corrections for significant deformations and movements, along with the incremental formulation of Hooke’s law, it is possible to integrate the shear stress τ over time using the strain rates.

(13) $$\frac{d\tau_{i}^{\alpha \beta }}{dt}=2G\left({\dot{\varepsilon }}_{i}^{\alpha \beta }-\frac{1}{3}\delta^{\alpha \beta }{\dot{\varepsilon }}_{i}^{\gamma \gamma }\right)+\tau_{i}^{\alpha \gamma }\cdot {\dot{r}}_{i}^{\beta \gamma }+\tau_{i}^{\gamma \beta }\cdot {\dot{r}}_{i}^{\alpha \gamma }.$$

*G* represents the shear modulus of a particular solid material, which can be determined through experimental procedures. On the other hand, the strain rate tensor denoted by ${\dot{\varepsilon }}^{\alpha \beta }$ establishes a connection between the derivatives of the velocity field:

(14) $${\dot{\varepsilon }}^{\alpha \beta }=\frac{1}{2}(\frac{\partial v^{\alpha }}{\partial x^{\beta }}+\frac{\partial v^{\beta }}{\partial x^{\alpha }}).$$

At particle *i*, the SPH formulations is as follows:

(15) $$\varepsilon_{i}^{\alpha \beta }=\frac{1}{2}\sum_{j=1}^{N}\left(\frac{m_{j}}{\rho_{j}}v_{ji}^{\alpha }\frac{\partial W_{ij}^{new}}{\partial x_{i}^{\beta }}+\frac{m_{j}}{\rho_{j}}v_{ji}^{\beta }\frac{\partial W_{ij}^{new}}{\partial x_{i}^{\alpha }}\right).$$

The rotation rate tensor ${\dot{r}}^{\alpha \beta }$ is given by

(16) $${\dot{r}}^{\alpha \beta }=\frac{1}{2}(\frac{\partial v^{\alpha }}{\partial x^{\beta }}-\frac{\partial v^{\beta }}{\partial x^{\alpha }}).$$

It can be also discretized in particle summation at particle *i* by the same SPH formulations:

(17) $$r_{i}^{\alpha \beta }=\frac{1}{2}\sum_{j=1}^{N}\left(\frac{m_{j}}{\rho_{j}}v_{ji}^{\alpha }\frac{\partial W_{ij}^{new}}{\partial x_{i}^{\beta }}-\frac{m_{j}}{\rho_{j}}v_{ji}^{\beta }\frac{\partial W_{ij}^{new}}{\partial x_{i}^{\alpha }}\right).$$

Once the elastic deformation of a material occurs, it transitions into plastic deformation. In this paper, the aforementioned elastic constitutive relationship can be expanded to encompass plastic behavior through the application of the von-Mises yield criterion, which is utilized to determine the critical point at which plastic deformation takes place [22].

(18) $$f_{y}=\frac{\sigma_{y}}{\sqrt{3J_{2}}}.$$

The flow stress represented by symbol $\sigma_{y}$ initially corresponds to the shear stress, denoted by $\sigma_{y}=\sqrt{\tau^{\alpha \beta }\cdot \tau^{\alpha \beta }}$. The second invariant of the stress tensor is given by $J_{2}$. Plastic deformation is said to occur when the value of $f_{y}$ is less than 1. At this point, the deviatoric stress components are returned to the yield surface and denoted by the $\tau^{\alpha \beta }=f_{y}\tau^{\alpha \beta }$ [19].

### 2.2. Material Constitutive

#### 2.2.1. Johnson–Cook Damage Material Constitutive law

The Johnson-Cook constitutive model [24] is employed to represent the behavior of the workpiece material during the cutting process, taking into consideration the impact of strain hardening, strain rate hardening, and thermal softening. In this model, the flow stress is calculated using

(19) $$\sigma_{y}=\left[A+B{\left(\varepsilon_{eff}^{p}\right)}^{N}\right]\left[1+Cln(\frac{{\dot{\varepsilon }}_{eff}^{p}}{{\dot{\varepsilon }}_{0}})\right]\left[1-{\left(\frac{T-T_{room}}{T_{melt}-T_{room}}\right)}^{M}\right],$$

where $\varepsilon_{eff}^{p}(=\int {\dot{\varepsilon }}_{eff}^{p}dt)$ is the equivalent plastic strain, ${\dot{\varepsilon }}_{eff}^{p}$ is the equivalent plastic strain rate, ${\dot{\varepsilon }}_{0}$ is the reference value of equivalent plastic strain rate, and *A*, *B*, *C*, *n,* and *m* are material dependent constants. $T_{room}$ is the room temperature, $T_{melt}$ is the melting temperature of the concerned solid material, and *T* is the current temperature and can be calculated using the following equation:

(20) $$e=\int C_{p}dT\approx C_{p}(T-T_{room}).$$

To describe local damage during chip formation by a cutting tool, a damage initiation criterion is employed using the JC damage initiation method [25], with the parameter *D* used to identify local damage conditions. The value of *D* is computed using the increment of equivalent plastic strain during an integration cycle and the strain to failure. The latter is given by a separate equation involving additional material-dependent constants and a ratio of pressure to the von Mises stress.

(21) $$D=\sum \frac{∆{\dot{\varepsilon }}_{eff}^{p}}{\varepsilon_{failure}},$$

where $∆{\dot{\varepsilon }}_{eff}^{p}$ is the increment of equivalent plastic strain occurring during an integration cycle, and $\varepsilon_{failure}$ is the strain to failure given by the following equation:

(22) $$\varepsilon_{failure}=\left[D_{1}+D_{2}\exp(D_{3}\sigma^{*})\right]\left[1+D_{4}ln(\frac{{\dot{\varepsilon }}_{eff}^{p}}{\dot{\varepsilon_{0}}})\right]\left[1+D_{5}(\frac{T-T_{room}}{T_{melt}-T_{room}})\right],$$

where $D_{1}\sim D_{5}$ are material dependent constants, $\sigma^{*}$ is the ratio of pressure to the von Mises stress.

(23) $$\sigma^{*}=\frac{P}{\sigma_{eff}}.$$

When the value of *D* reaches 1 at a specific particle, it signifies that the material structure at that point has been destroyed by cutting and that material failure has occurred. The corresponding stress components at that particle i are then reset to zero and remain so during subsequent analysis. Although the JC damage law is capable of modeling the saw-tooth shape observed during cutting process simulations, it does not account for strain-softening or dynamic recrystallization mechanisms. All parameters are given in Table 1. $\rho_{0}$ is the initial density which is used to calculate pressure *P* (Equation (11)). *T_room_* is the room temperature, *T_melt_* is the melting temperature of the concerned solid material. *T* is the current temperature. They are used to calculate flow stress $\sigma_{y}$ in both model JC and JC-TANH (Equations (19) and (24)). Specific heat *C_p_* is used to calculate the internal energy per unit of mass e (Equation (20)). *A*, *B*, *C*, *n,* and *m* are material-dependent constants of model JC, and $D_{1}\sim D_{5}$ are material-dependent constants of the JC damage model.

#### 2.2.2. Modified Constitutive Law-TANH

A developed model called TANH has been successfully incorporated into the Finite Element Method (FEM) code to introduce the strain softening effect [34,36,37]. This model is based on a modified JC law and considers the influence of strain, strain rate, and temperature on the flow stress. By implementing this constitutive model into our SPH code, the new material flow stress can be expressed using the equation below:

(24) $$\sigma_{y}=\left[A+B{\left(\frac{1}{{\exp(\varepsilon }_{eff}^{a})}\right)}^{N}\right]\left[1+Cln\left(\frac{{\dot{\varepsilon }}_{eff}^{p}}{{\dot{\varepsilon }}_{0}}\right)\right]\left[1-{\left(\frac{T-T_{room}}{T_{melt}-T_{room}}\right)}^{M}\right]{\left[\left(D+(1-D)\tanh(\frac{1}{{\left(\varepsilon +S\right)}^{c}}\right)\right]}^{r},$$

where

(25) $$D=1-{\left(\frac{T}{T_{m}}\right)}^{d},$$

(26) $$S={\left(\frac{T}{T_{m}}\right)}^{b}.$$

**Figure 5:**
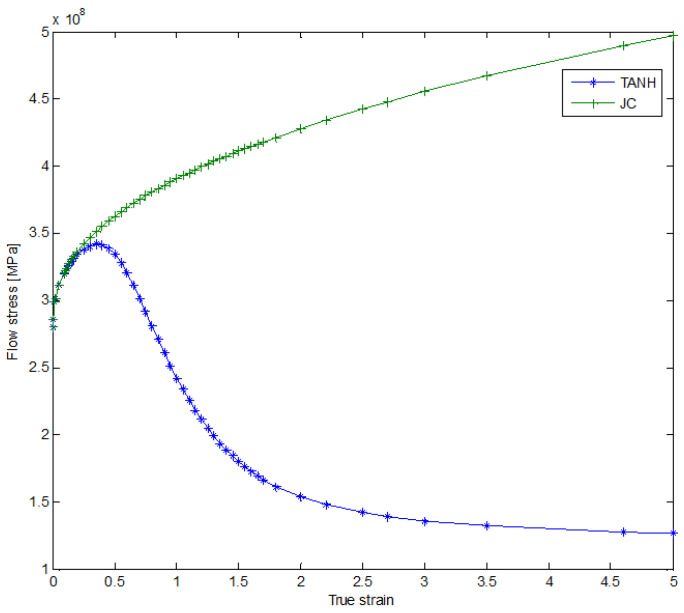
Comparison between for JC law and TANH (flow stress–strain curves).

The equation for the new material flow stress includes parameters *A*, *B*, *C*, *n*, and *m*, which are the same as those used in the JC equation. These parameters can be determined through Split Hopkinson Pressure Bar (SHPB) tests. Additionally, there are corrected parameters *a*, *b*, *c*, *d* and *r*. According to the TANH model, at low strains, the flow stress increases in a manner similar to that predicted by the JC constitutive law, as shown in Figure 5. However, beyond a certain strain, the flow stress begins to decrease until it reaches a strain of approximately 3, after which the flow stress remains nearly constant. This behavior is associated with the strain-softening and dynamic recrystallization processes that occur in materials.

In order to identify parameters *a, b, c, d* and *r*, their individual effects on the strain–stress curves are studied by varying one parameter while keeping the others constant. The parameters are selected such that the resulting strain–stress curve is in good agreement with both the TANH and JC model curves under low strain conditions [26]. By analyzing the trends of the curves with different parameters, the best-fit values are determined and presented in Table 2.

#### 2.2.3. Joint JC-TANH Law

**Figure 6:**
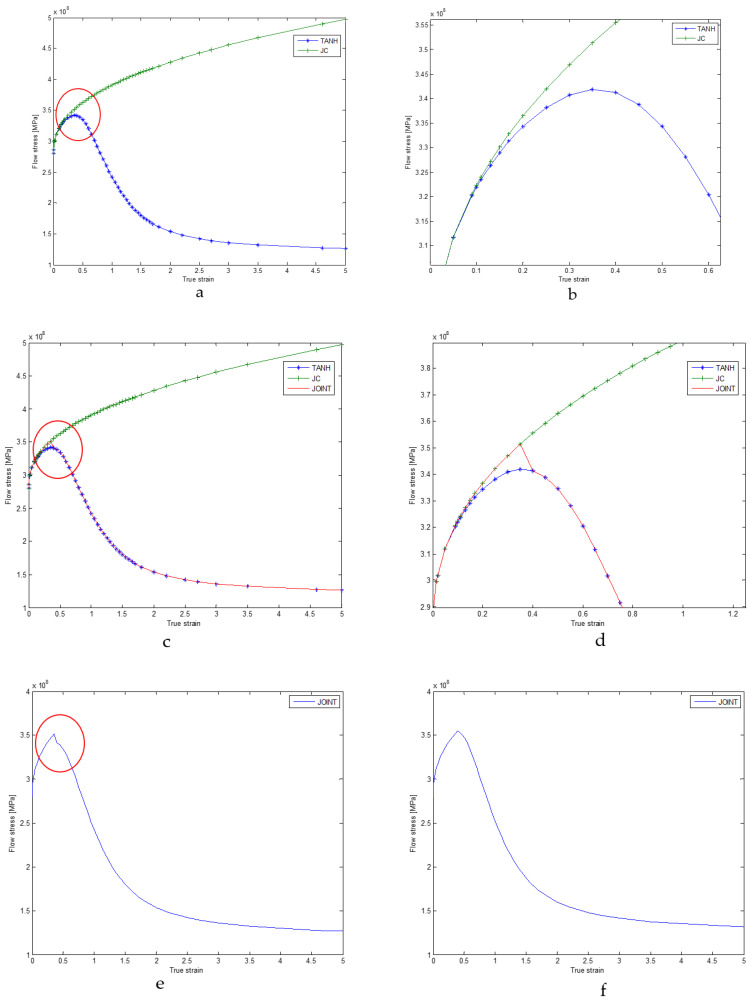
Stress–strain curve of JC-TANH constitutive model ((**a**) represents the stress-strain curves difference between the JC and TANH model; (**b**) represents the two model difference in red circle area; (**c**) represents the stress-strain curve difference between the joint model and other two model; (**d**) represents the three model difference in red circle area; (**e**) represents the joint JC-TANH model stress-strain curve, (**f**) represents modified joint model stress-strain curves).

The JC law can express the material behavior well when the strain is under low strain, which can account for the effects of strain hardening, strain rate hardening and thermal softening. However, it cannot describe the strain-softening process. Though the TANH can describe the strain softening, it cannot accurately describe the strain-hardening process as well as JC, because the TANH model changes the equation of JC laws (Figure 6a), and there is still a difference between JC and TANH in describing strain hardening, strain rate hardening and thermal softening (Figure 6b). In order to reduce this difference, we separate this process into two parts after the flow stress reaches the yield point, which means we can describe the material behavior by two equations (JC equation and TANH equation). When the strain is under coupling node, we adopt the JC law to describe the first process (strain hardening, strain rate hardening and thermal softening), and when the strain is beyond coupling node, we employ the TANH law to describe the strain-softening process (Figure 6c,d). By integrating the two laws, we avoid the time-consuming process of fitting the TANH law to the JC law in the strain-hardening part, and we assure the accuracy of the modeling flow stress by applying the JC law under the low-strain condition. It is very important to search the coupling node of the JC model and the TANH model. This requires us to carry out hyperbolic similarity evaluation and search for strain nodes that can make the JC and TANH curves reach the highest similarity. The curve similarity evaluation formula is as follows:

(27) $$S\left(a,b,c,d,r\right)=\int_{0}^{e}\left|\sigma_{JC}-\sigma_{TANH}\right|d,$$

where $\sigma_{JC}$ is the Johnson–Cook constitutive flow stress, which can be calculated by Equation (19), $\sigma_{TANH}$ is thr TANH constitutive flow stress, which can be calculated by Equation (24), *e* is the scope of strain, which can be set to 10, and *S* is hyperbolic similarity evaluation. The smaller the value of *S*, the greater the similarity between the two curves.

Here, we use the GA algorithm to search for *a*, *b*, *c*, *d* and *r,* looking for a set of numbers that can make the function minimum. To reduce calculation complexity, the values of *a*, *b*, *c*, *d* and *r* are set to a range from 0 to 100. The specific algorithm steps are shown in the following list:

Step 1 Set the following parameters: Population size: 200, crossover probability: 0.7, mutation probability: 0.03, maximum number of iterations: 2000.
Step 2 Initial population: Generate initial population randomly. Each individual in the population is a solution, that is, a combination of variables encoded by the real numbers;
Step 3 Calculate the fitness of the individual: Use an objective function S(a,b,c,d,r) to calculate the fitness of each individual.
Step 4 Check whether the end conditions are met. If an end condition is met (for example, a preset maximum number of iterations is reached, or the optimal fitness is no longer significantly improved), the algorithm is terminated and the current optimal solution is output. Otherwise, proceed to the selection operation.
Step 5 Selection: Select according to the fitness of the individual. Individuals with higher fitness have a higher probability of being selected.
Step 6 Cross: A cross-operation is performed on the selected individual to generate a new individual.
Step 7 Variation: Variation is performed on the newly generated individuals to increase the diversity of the population.
Step 8 Assess the fitness of each individual in a new population: Assess the fitness of each individual in a new population using the same method.
Step 9 Return to Step 4 and iterate until the end condition is met.

The parameters of the TANH model with the least similarity are presented in Table 2. The coupling node of the strain of the two models is 0.3. In order to make the connection between the TANH law and the JC law smooth, we adjust parameter *a* (Figure 6f). This process only involves one parameter instead of several parameters which are required when using the TANH law to fit JC in the strain-hardening part. To summarize, the joint constitutive model has two advantages compared to the TANH model: (1) It is more accurate when describing the relationship between strain and stress under the low-strain condition; (2) It does not need to fit the JC curve at the early stage of strain changes.

In addition to this, in this study, an effective and useful contact algorithm was utilized in the SPH code [26]. When the cutting tool particles and workpiece particles come close to each other, an interaction occurs.

## 3. Experiment Results and Discussion

**Figure 7:**
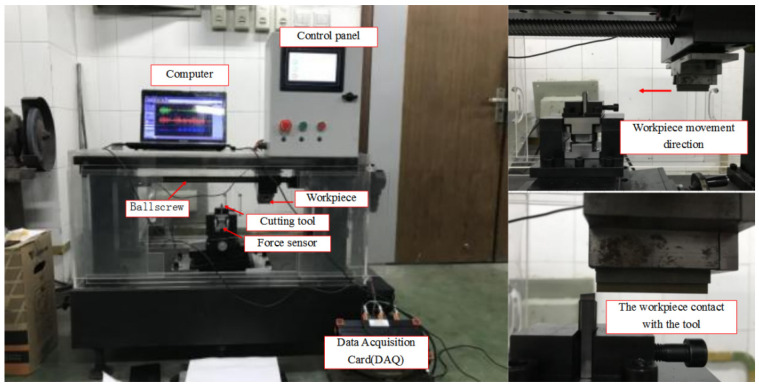
Experimental setup for cutting test.

**Figure 8:**
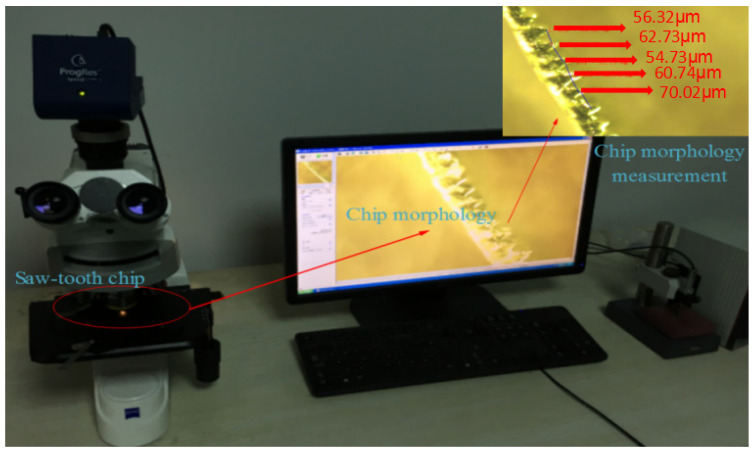
Experimental chip morphology measurement.

In this experimental test, the rake angle of the cutter is 0°. The tip radius of the cutter is 20 μm. The experimental platform mainly uses the ball screw to convert the rotation of the motor into a transverse motion. The table is installed on the ball screw, and the workpiece is clamped on the workbench to achieve orthogonal cutting. At the same time, in order to better measure the size of the cutting force in the cutting process, the force sensor is installed under the tool which uses a special fixture; the tool is made of a customized cemented carbide non-standard tool, and the tool shape is similar to that of a planer tool. Experimental condition and equipment are as shown in Figure 7 and Figure 8.

**Figure 9:**
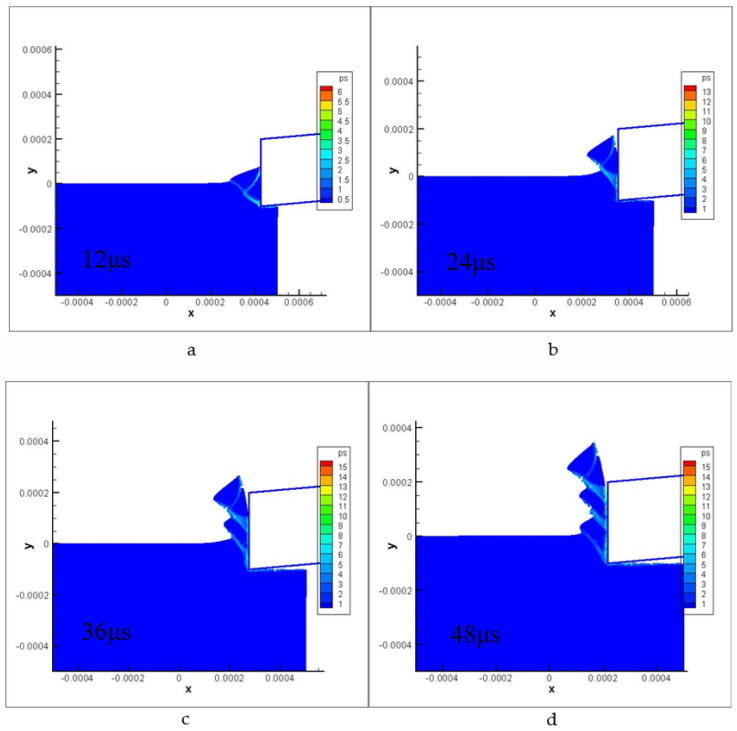
Plastic strain of localized chip formation steps. ((**a**–**d**) represent the strain change and chip shape of the model at different times in the cutting process).

**Figure 10:**
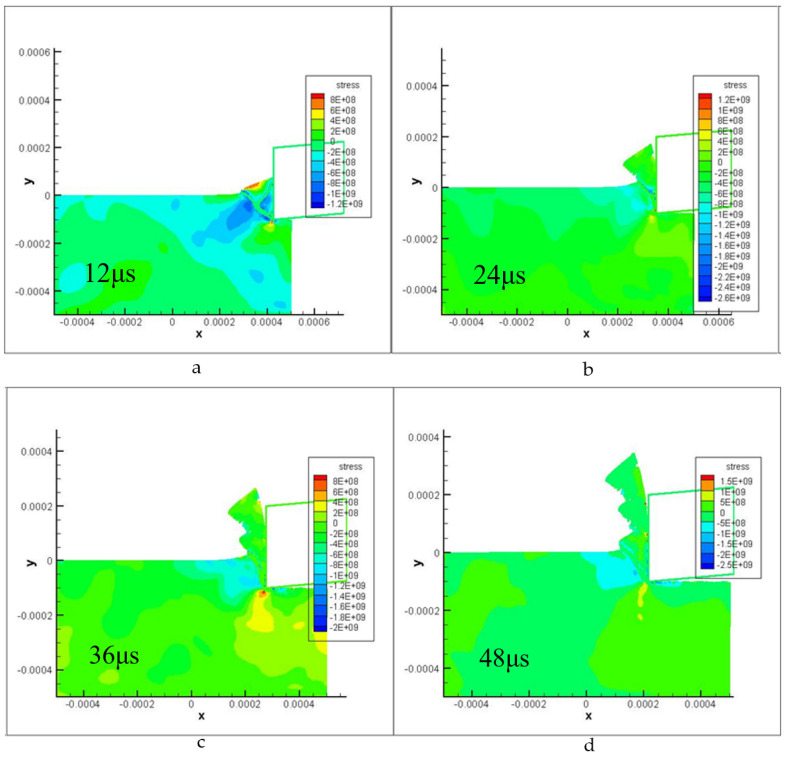
Shear stress of localized chip formation steps.((**a**–**d**) represent the stress change and chip shape of the model at different times in the cutting process).

Figure 9 and Figure 10 display the contours of plastic strain and shear stress during the saw-tooth chip deformation process at various times. The cutting parameters used in the simulations were a cutting depth of 0.1 mm and a cutting speed of 235 m/min. The simulation results demonstrate excellent agreement with the actual experimental results, revealing strain localization along a curved shear and the curvature changes in the slipped shear band (new segment). These findings indicate that the simulation model accurately captures the complex deformation behavior of saw-tooth chips during the cutting process.

**Figure 11:**
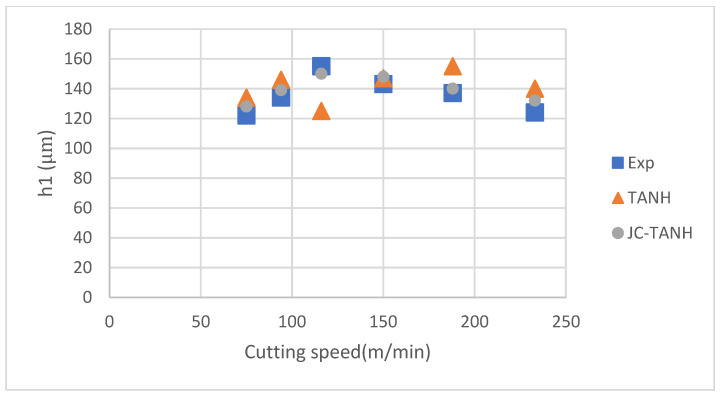
Maximum segmentation height (h1) for different cutting speeds with different constitutive laws.

**Figure 12:**
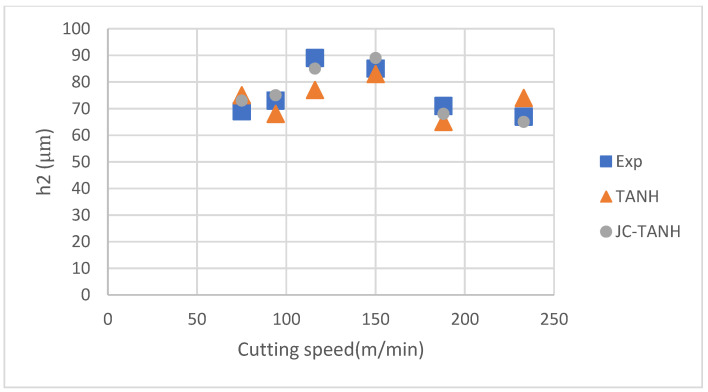
Minimum segmentation height (h2) for different cutting speeds with different constitutive laws.

**Figure 13:**
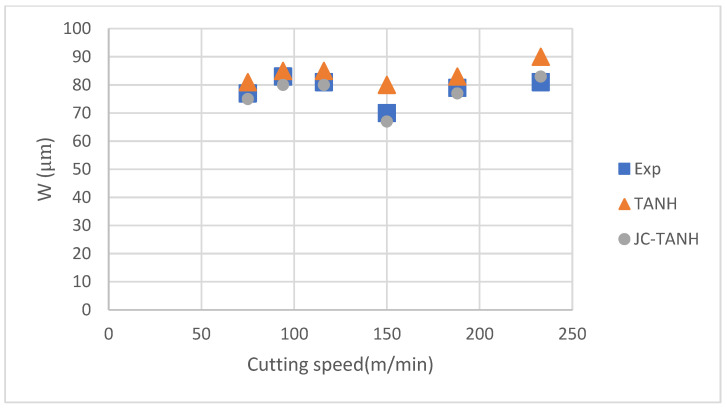
Segmentation length (W) for different cutting speeds with different constitutive laws.

**Figure 14:**
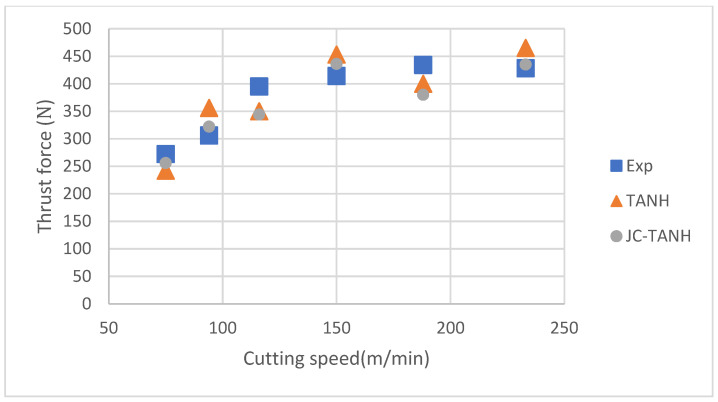
Average thrust force under different cutting speeds.

**Figure 15:**
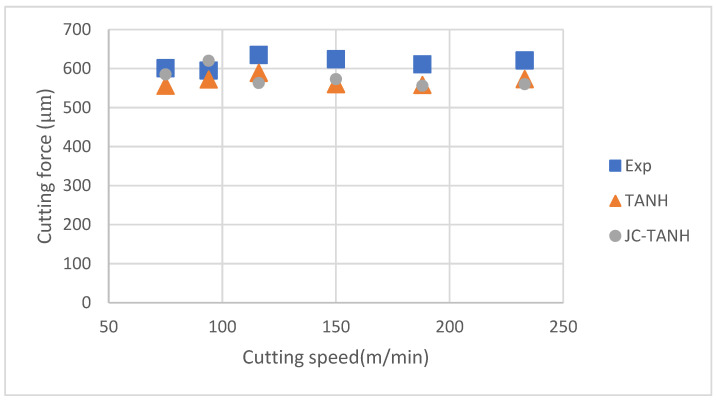
Average cutting force under different cutting speeds.

We conducted a comparative analysis of two simulation results and experimentally observed chip morphology to identify differences among the three different laws. The segmented chip morphology was characterized by using three parameters: segmentation length (W), maximum segmentation height (h1), and minimum segmentation height (h2) (Figure 1b). We quantitatively compared the simulation results from TANH and JC-TANH with the experimental chip morphology and cutting force. The morphology comparisons are presented in Figure 11, Figure 12 and Figure 13, while the cutting force comparisons are illustrated in Figure 14 and Figure 15. Our findings demonstrate that the JC-TANH law provides better simulation results compared to the TANH law. Table 3 and Table 4 present the quantitative differences in relative errors between the two simulations and the experimental result at different cutting speeds. The results reveal that the relative errors of the JC-TANH law are lower than those of the TANH law.

**Figure 16:**
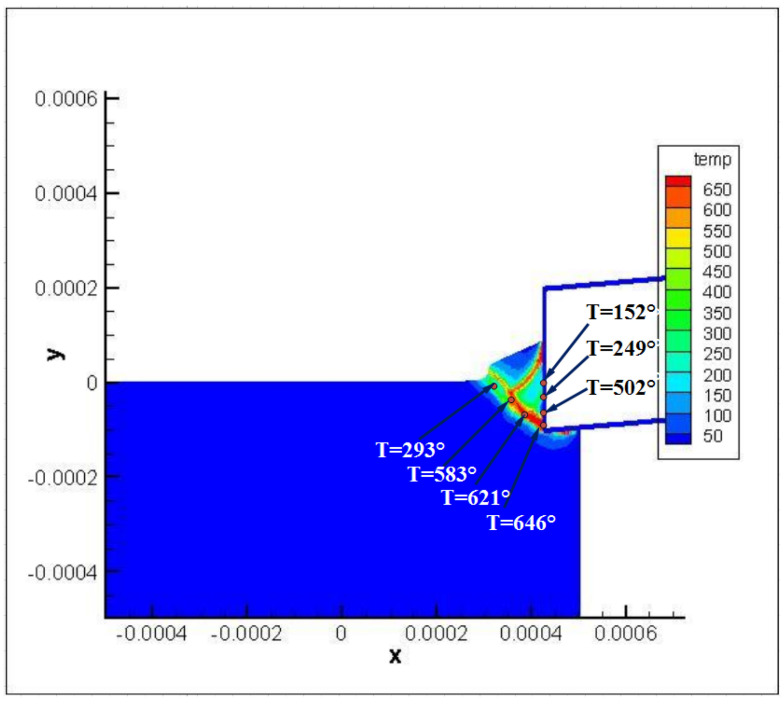
The temperature distribution near the cutting tool tip.

Figure 16 shows the temperature distribution near the cutting tool tip when a new segmented chip forms. From Figure 15 we can see that the temperature rises rapidly in the main shear zone, and the closer the point position, the higher the temperature. The temperatures along the tool rake face are far from being homogeneous, and the thermal softening effect also occurs during this process.

**Figure 17:**
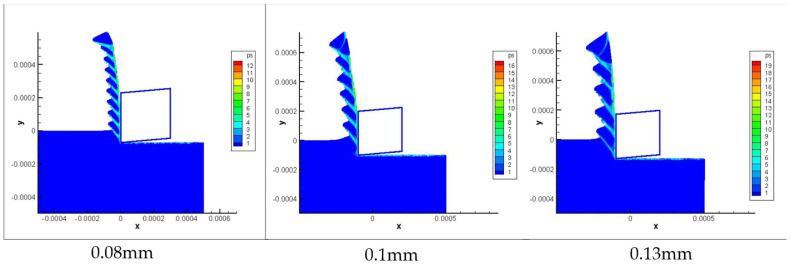
The chip morphology with different cutting depths.

**Figure 18:**
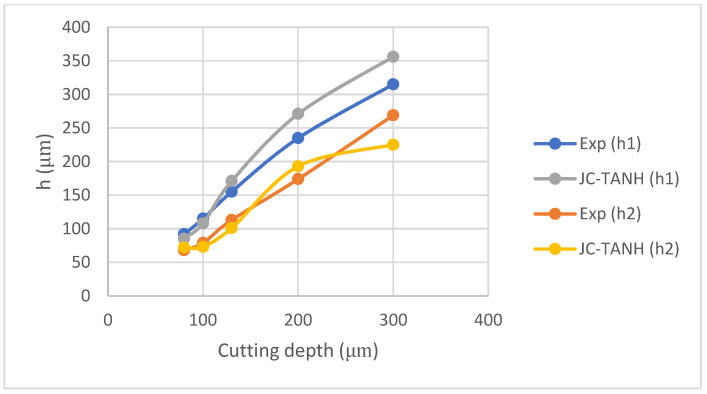
Maximum segmentation height (h1) and minimum segmentation height (h2) for different cutting depths with JC-TANH constitutive law.

**Figure 19:**
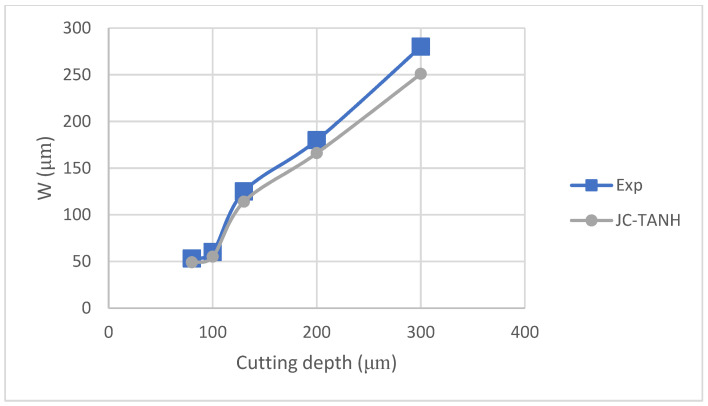
Segmentation length (W) for different cutting depths with JC-TANH constitutive law.

Figure 17 is the chip morphology simulation with the JC-TANH law for different cutting depths. Figure 18 and Figure 19 present comparisons of the chip morphology parameters with different cutting depths between simulation and experimental results. Table 5 shows the comparisons of absolute and relative errors in simulations and experimental results for chip morphology parameters.

From Figure 18 and Figure 19 and Table 5, we conclude that the JC-TANH law can also predict the chip morphology with different cutting depths accurately. It should be noted that the predicted errors of chip morphology increase as the cutting depth increases, since the vibration of the machine tool intensifies with the increase in cutting depth, and the intensification affects the saw-tooth formation and leads to the differences between the experimental results and simulations.

## 4. Conclusions

The accuracy of cutting simulation is dependent on the selection of the constitutive law, which is crucial in predicting the chip morphology, cutting forces, and thrust forces with precision. In this research, an enhanced SPH method was used to implement a new joint constitutive model aimed at improving the cutting model. The experiments conducted using different constitutive laws (TANH and JC-TANH) indicate that the new joint constitutive law (JC-TANH) provides the best agreement with the experimental outcomes.

From Table 3, the maximum relative error of h1 of the JC-TANH model for the prediction of chip shape is 7.19%, which is significantly lower than the 19.35% of the TANH model. Similarly, the maximum relative error of h2 of the JC-TANH model is 8.99%. The relative error of TANH model is as high as 13.48%, the maximum relative error of the JC-TANH model W is 7.47%, and the maximum relative error of the TANH model is 11.11%. It can be proved by these data that the joint JC-TANH constitutive cutting model improves the prediction of chip morphology and simulates the process of saw-tooth chip formation. In the simulation of the cutting process based on the SPH method, we did not find the calculation interruption due to a large deformation problem; the model also does not require specific treatments such as adaptive re-meshing, deleting elements, or setting separate chip criteria in distorted computational domains. In addition, throughout the cutting process, chip morphology and distinct shearing band can be modeled clearly as the experiment observes, which verifies the hypothesis that the joint constitutive law is effective in simulating the cutting process of Ti-6AL-4V. In conclusion, the improved SPH cutting model has the following advantages:

1. The SPH cutting model possesses a significant advantage in dealing with large deformations and the simulation process is more consistent with the actual cutting process;
2. Chip morphology and distinct shearing band can be modeled clearly;
3. The joint JC-TANH constitutive cutting model improves the prediction of chip morphology.

## Figures and Tables

**Table 1:** Material properties of Ti-6AL-4V for workpiece [33,34,35].

Physical Parameter		Work Material (Ti-6AL-4V)
Density $\rho_{0}\;(kg/m^{3})$		4430
Possion’s ratio *v*		0.342
Specific heat $C_{p}$ J/(kgK)		580
Thermal conductivity (W/mk)		7.3
$T_{melt}$ (°C)		1878
$T_{room}$ (°C)		25
Johnson–Cook plasticity model (Equation (19)) [34]	*A*	968 MPa
	*B*	380 MPa
	*C*	0.02
	*n*	0.577
	*m*	0.421
Johnson–Cook Damage model (Equation (22)) [35]	*D*1	−0.09
	*D*2	0.25
	*D*3	−0.5
	*D*4	0.014
	*D*5	3.87

**Table 2:** TANH constitutive model parameters of Ti-6AL-4V for workpiece.

TANH model (Equation (24))	*a*	0.05
	*b*	2
	*c*	1
	*d*	1
	*r*	5

**Table 3:** Comparison of errors in simulation and experimental data for segmentation characteristics.

Speed	h1 (Relative Error %)		h2 (Relative Error %)		W (Relative Error %)	
	TANH	JC-TANH	TANH	JC-TANH	TANH	JC-TANH
80	9.84%	4.92%	8.70%	5.80%	5.19%	−4.60%
100	8.96%	3.73%	−6.85%	5.74%	2.41%	−3.61%
120	−19.35%	−7.23%	−13.48%	−4.49%	4.94%	−1.23%
150	2.80%	3.50%	−2.35%	4.71%	14.29%	−4.29%
180	13.14%	7.19%	−8.45%	−4.23%	5.06%	−4.53%
230	12.90%	6.45%	10.45%	−8.99%	11.11%	7.47%

**Table 4:** Comparison of errors in simulation and experimental data for cutting force and feed force values.

Speed	Cutting Force (Relative Error %)		Thrust Force (Relative Error %)	
	TANH	JC-TANH	TANH	JC-TANH
80	−7.49%	−2.66%	−11.03%	−5.88%
100	−3.87%	4.20%	16.34%	5.23%
120	−7.24%	−11.34%	−11.39%	−12.91%
150	−10.26%	−8.17%	9.42%	5.31%
180	−8.67%	−9.00%	−7.83%	−12.44%
230	−7.73%	−9.82%	8.64%	3.64%

**Table 5:** Comparison of errors in simulation and experimental data for chip morphology parameters.

Cutting Depth	h1		h2		W	
	Absolute Error (μm)	Relative Error (%)	Absolute Error (μm)	Relative Error (%)	Absolute Error (μm)	Relative Error (%)
0.08	−7	−7.61%	4	5.88%	−4	−7.55%
0.1	−7	−6.09%	−6	−7.59%	−5	−8.33%
0.13	16	10.32%	−12	−10.62%	−11	−8.80%
0.2	36	15.32%	19	10.92%	−14	−7.78%
0.3	41	13.02%	−44	−16.36%	−29	−10.36%

## Data Availability

Not applicable.
